# Development and validation of stable ferroptosis‐ and pyroptosis‐related signatures in predicting prognosis and immune status in breast cancer

**DOI:** 10.1111/jcmm.17958

**Published:** 2023-10-18

**Authors:** Lili Zhou, Chinting Wong, Yang Liu, Wenyan Jiang, Qi Yang

**Affiliations:** ^1^ Department of Radiology The First Hospital of Jilin University Changchun China; ^2^ Department of Nuclear Medicine The First Hospital of Jilin University Changchun China

**Keywords:** breast cancer, ferroptosis, immune status, prognosis, pyroptosis

## Abstract

To develop and validate the predictive effects of stable ferroptosis‐ and pyroptosis‐related features on the prognosis and immune status of breast cancer (BC). We retrieved as well as downloaded ferroptosis‐ and pyroptosis‐related genes from the FerrDb and GeneCards databases. The minimum absolute contraction and selection operator (LASSO) algorithm in The Cancer Genome Atlas (TCGA) was used to construct a prognostic classifier combining the above two types of prognostic genes with differential expression, and the Integrated Gene Expression (GEO) dataset was used for validation. Seventeen genes presented a close association with BC prognosis. Thirteen key prognostic genes with prognostic value were considered to construct a new expression signature for classifying patients with BC into high‐ and low‐risk groups. Kaplan–Meier analysis revealed a worse prognosis in the high‐risk group. The receiver operating characteristic (ROC) curve and multivariate Cox regression analysis identified its predictive and independent features. Immune profile analysis showed that immunosuppressive cells were upregulated in the high‐risk group, and this risk model was related to immunosuppressive molecules. We successfully constructed combined features of ferroptosis and pyroptosis in BC that are closely related to prognosis, clinicopathological and immune features, chemotherapy efficacy and immunosuppressive molecules. However, further experimental studies are required to verify these findings.

## 1 INTRODUCTION

Breast cancer (BC) affects the breast tissue and can easily metastasize to the lungs and bones. BC has a high worldwide incidence.^1^ It is the second most common cause of death in females,^2^ accounting for approximately 24% of the confirmed cases and 15% of death cases.^3^ However, BC can only be diagnosed at a later stage because it is asymptomatic at an early stage, resulting in irreversible consequences. Therefore, early diagnosis and intervention are critical.^4^ Genome and transcriptome sequences have been used to divide BC into four intrinsic molecular subtypes: Luminal A/B, HER‐2 enrichment, basal‐like and claudin‐low.^5^ Although advances have been made in terms of diagnosis, surgery, chemotherapy and radiotherapy, BC still presents a high malignancy rate, and survival outcomes are poor.^6^ Researchers have found that BC is tolerant to certain antitumor drugs that exert apoptotic effects on tumour cells.^7^ Therefore, other possible forms of cell death should be evaluated to overcome chemotherapy resistance and identify novel biomarkers and treatment targets for improvement in the prognosis of BC.

Programmed cell death (PCD) is a process in which cells actively die after receiving certain signals or are stimulated by specific factors, to stabilize the internal environment. PCD can be observed not only during the normal development of individuals but also in abnormal physiological states or diseases.^8^ Recent studies have confirmed two new PCD modes, ferroptosis and pyroptosis, which exhibit remarkably different cell death pathways and relevant biochemical and morphological features from those of other cell death modes, such as apoptosis, necrosis and autophagy. Studies have shown that a variety of programmed cell death patterns play important roles in tumour progression and have the potential to become indicators of postoperative prognosis and drug sensitivity in BC.^9^

Ferroptosis refers to iron‐dependent regulatory cell death in which lipid peroxidation accumulates to a lethal level.^10^ Currently, three categories of genes take charge of regulating ferroptosis: ferroptosis (DOF), inhibitors of ferroptosis (SOF) and others that drive or inhibit ferroptosis, depending on the environment.^11^ Studies have shown that ferroptosis‐related marker genes can be used as new biomarkers to predict the prognosis of BC patients.^12^ Pyroptosis refers to the lytic form of regulatory cell death that releases many proinflammatory mediators. Death cell‐activated pyroptosis acts in two ways: GSDMD‐dependent under the regulation of Caspase1/4/5/11, and GSDME‐dependent under the regulation of Caspase 3.^13^ DRD2 regulates the microenvironment by promoting M1 polarization of macrophages and triggering pyroptosis to inhibit the occurrence and development of BC.^14^

The induction of ferroptosis and pyroptosis can improve anticancer immunity and inhibit tumour growth. However, the number of studies that have conducted systematic investigations on the possibility of combining these two cell death modes in BC remains limited. Therefore, this study first downloaded and screened ferroptosis and pyroptosis‐related genes from the database and then performed survival analysis, established a prognostic risk model, performed immune infiltration‐related analysis and finally validated the data in the Synthesis of Gene Expression (GEO) database to explore the predictive effects of ferroptosis and pyroptosis‐related characteristics on the prognosis and immune status of BC patients.

## 2 METHODS

### 2.1 TCGA and GEO data download

**FIGURE 1:**
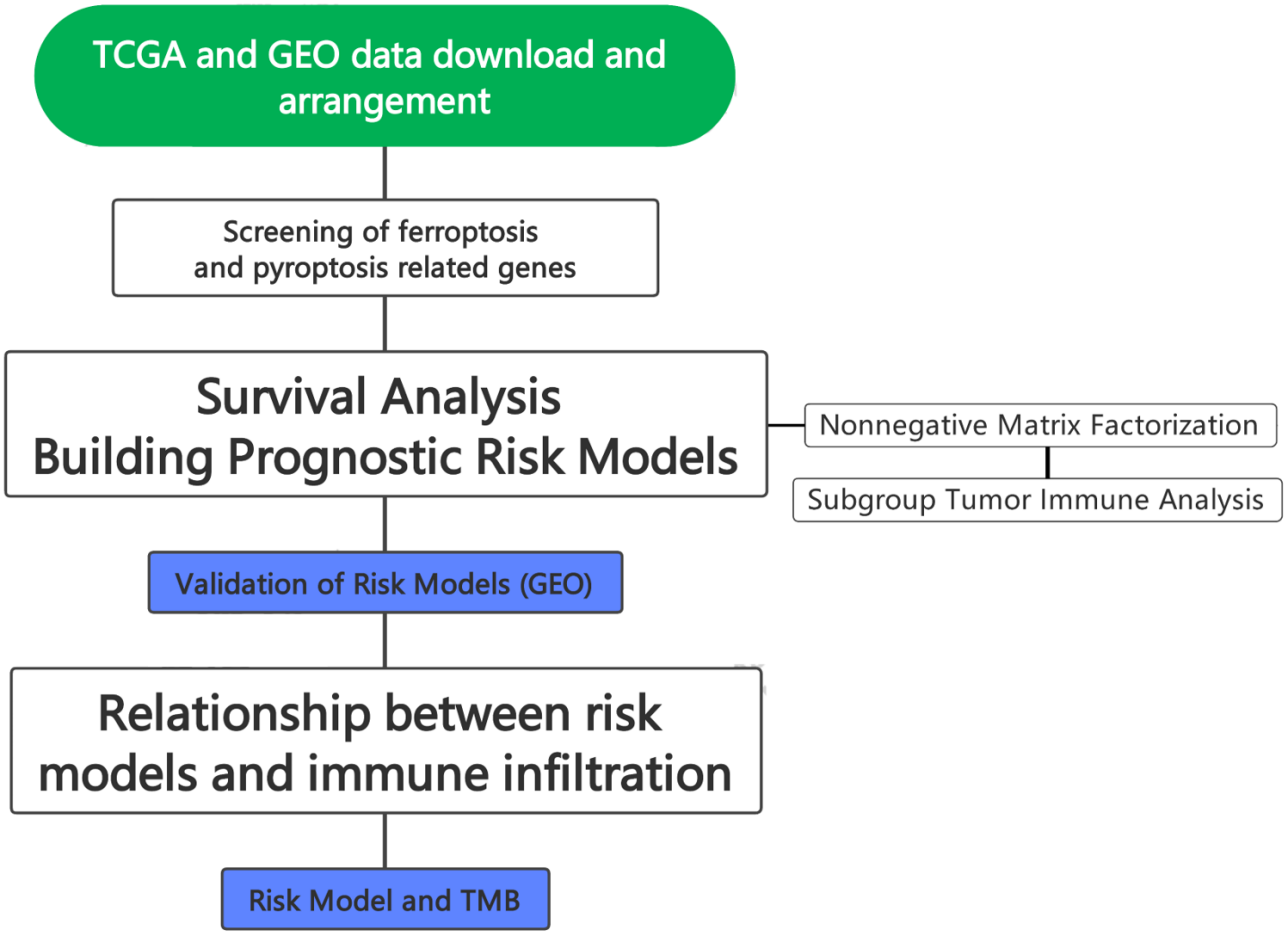
The flow chart.

We downloaded the mRNA expression data and clinical information of two independent BC cohorts from two public databases: The Cancer Genome Atlas (TCGA) (https://portal.gdc.cancer.gov/) (TCGA‐BC, *n* = 1098) and GEO (https://www.ncbi.nlm.nih.gov/geo/) (GSE42568, *n* = 121). The guidelines for the use of the TCGA and GEO databases are in line with the data collection requirements. We removed normal samples, TCGA‐BC patients with a survival of <30 days, missing survival time, missing stage information and male patients and obtained a matrix of 834 samples. For GEO data processing, we deleted normal samples, and the remaining data met the requirements. The ComBat function of the R ‘SVA’ package^15^ assisted in eliminating the batch effect in different data sets. We developed a flowchart (Figure 1) for a systematic description of the study.

### 2.2 Search of genes related to ferroptosis and pyroptosis

We obtained relevant studies from GeneCards, the Molecular Feature Database (MSigDB), FerrDb^16^ and those published previously (Table 1).

**TABLE 1:** Ferroptosis and pyroptosis genomes.

The genome	Gene
Ferroptosis gene	ACSL4, AKR1C1, AKR1C2, AKR1C3, ALOX15, ALOX5, ALOX12, ATP5MC3, CARS, CBS, CD44, CHAC1, CISD1, CS, DPP4, FANCD2, GCLC, GCLM, GLS2, GPX4, GSS, HMGCR, HSPB1, CRYAB, LPCAT3, MT1G, NCOA4, PTGS2, RPL8, SAT1, SLC7A11, FDFT1, TFRC, TP53, EMC2, AIFM2, PHKG2, HSBP1, ACO1, FTH1, STEAP3, NFS1, ACSL3, ACACA, PEBP1, ZEB1, SQLE, FADS2, NFE2L2, KEAP1, NQO1, NOX1, ABCC1, SLC1A5, GOT1, G6PD, PGD, IREB2, HMOX1, ACSF2
Pyroptosis gene	AIM2, CA3, CASP1, CASP3, CASP4, CASP5, CASP6, CASP8, CASP9, ELANE, GSDMA, GSDMB, GSDMC, GSDMD, GSDME, IL18, IL1B, IL6, NLRC4, NLRP1, NLRP2, NLRP3, NLRP6, NLRP7, NOD1, NOD2, PJVK, PLCG1, PRKACA, PYCARD, SCAF11, TIRAP, TNF

### 2.3 Identification of BC subclasses

The genes related to ferroptosis and pyroptosis were used for nonnegative matrix factorization (NMF) clustering before the filtering procedure. We excluded candidate genes with MAD ≤ 0.5 in BC patients. MAD serves to measure statistical bias, provides a more reliable measure of variance relative to the standard deviation, is capable of better accommodating outliers in the dataset, and the final result cannot be affected by fewer outliers. The unsupervised NMF clustering method was performed on the metadata set using the NMF R package, and the optimal number of clusters was chosen as the coexistence correlation coefficient K value of 3.

### 2.4 Establishment of a risk score for predicting BC OS in a training cohort

Univariate Cox regression analysis was used to identify overall survival (OS) OS‐associated genes in the training cohort (*n* = 843), with a *p*‐value < 0.05. LASSO algorithm performed penalty parameter adjustment through 10‐fold cross‐validation via R package, for eliminating overfitting among prognostic genes and reducing the range of prognostic genes ‘Glmnet’. We included genes with non‐zero regression coefficients according to the LASSO regression analysis in the multivariate Cox regression analysis. The risk score was the corresponding regression coefficient obtained by multiplying the expression of each gene with the multivariate Cox regression analysis of each gene.^17^ The median risk score (MRS) was used to divide patients into high‐ and low‐risk groups. Kaplan–Meier survival curves as well as the time‐dependent receiver operating characteristic (ROC) obtained using the R packages ‘SuvMiner’ and ‘Survival ROC’, served for evaluating the predictive effect of risk score on BC patients' OS. Statistical significance was set at *p* < 0.

### 2.5 Evaluation of the tumour immune microenvironment using the ssGSEA algorithm

The single‐sample gene set enrichment analysis (ssGSEA) algorithm was used to quantify the infiltration abundances of 22 immune cell types using the normalized enrichment score (NES). An independent sample *t*‐test was used to compare the infiltration abundance between the two groups, *p* < 0.05 was considered statistically significant.

### 2.6 Exploration of the genomic landscape and stemness exhibited by prognostic features

We compared the two groups in terms of gene mutation rate and tumour mutation burden (TMB) using somatic mutation data from the TCGA GDC Data portal. mRNAsi is a quantitative index that reflects the features exhibited by cancer stem cells and is calculated based on gene expression data. A value closer to 1 indicated a lower degree of cell differentiation and stronger stem cell characteristics. Independent sample T was used to compare the two groups, and the Spearman correlation test was used to analyse the association between the risk score and TMB. Statistical significance was set at *p* < 0.05.

### 2.7 Statistical analysis

R (version 4.2.1) and SSPS (SPSS Inc.) were used for statistical analyses. The identification of survival‐related genes relied on univariate Cox regression analysis together with survival analysis, and Kaplan–Meier curves were used to identify these genes. Multivariate COX regression analysis was used to screen prognostic predictive genes for prognostic model construction. Time‐dependent ROC analysis was used to assess the predictive effects of the model. Student's *t*‐test and one‐way analysis of variance (anova) were used for statistical analysis between two groups and multiple groups, respectively. Statistical significance was set at *p* < 0.05.

## 3 RESULTS

### 3.1 Relation between the subtypes of ferroptosis and pyroptosis and BC prognosis, and prognosis prediction model establishment

**FIGURE 2:**
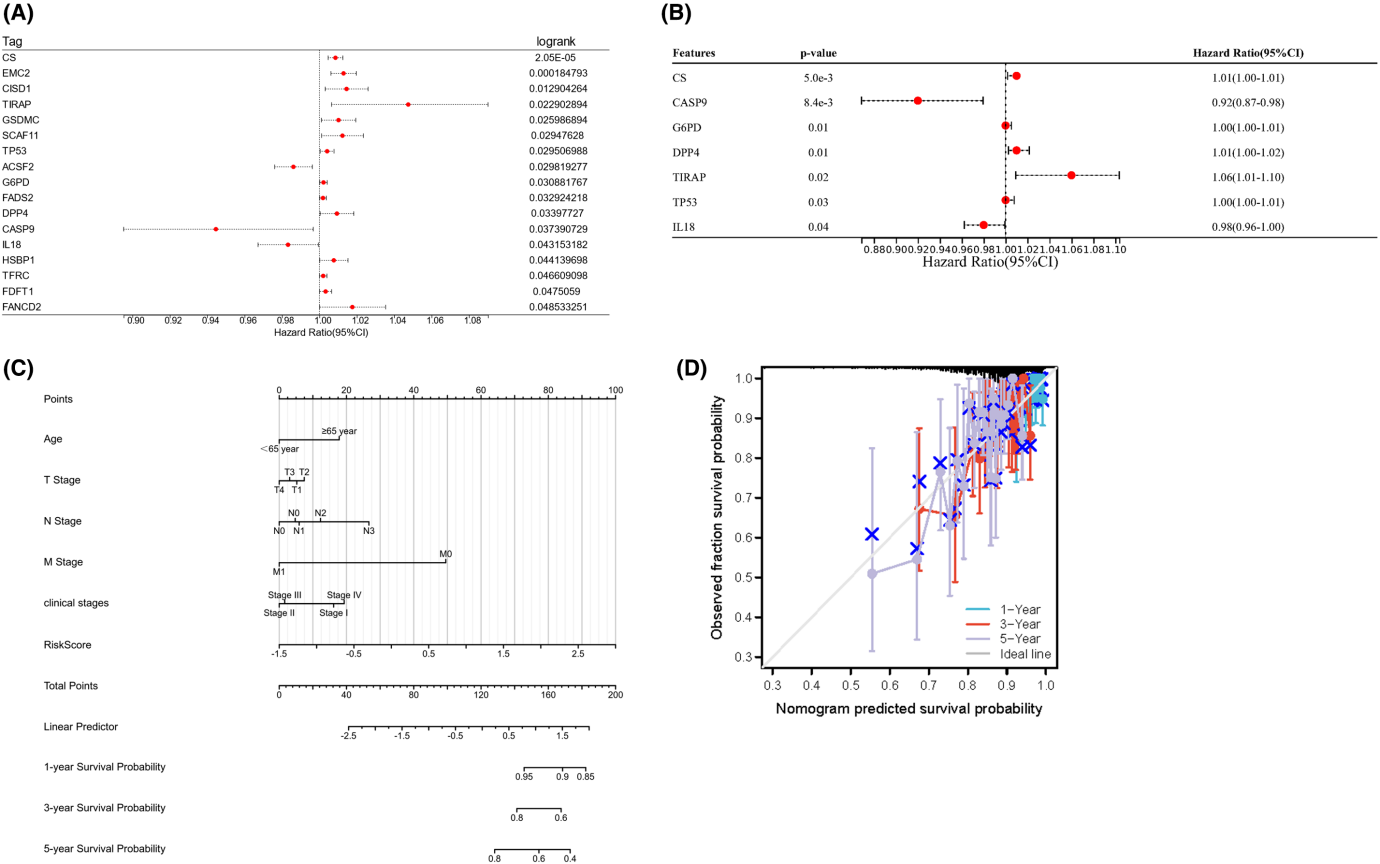
Relationship between ferroptosis and pyroptosis gene in breast cancer survival. (A) Univariate Cox regression analysis was used to analyse the relationship between ferroptosis and pyroptosis gene in breast cancer survival. (B) Multivariate analysis of TCGA breast cancer. (C) NOMO diagram of TCGA breast cancer. (D) NOMO survival curve correction.

Univariate Cox regression analysis confirmed that 17 genes were associated with OS in BC patients (Figure 2A). Using multivariate Cox regression, we found that CS, CASP9, G6PD, DPP4, TIRAP, TP53 and IL‐18 independently predicted the prognosis of patients with BC (Figure 2B). A nomogram can effectively help to assess individual risks in clinical settings during the quantification of multiple risk factors. We obtained a nomogram combining clinical data and risk scores, predicting the three‐ and five‐year OS using a synthesis of seven ferroptosis‐related genetic signatures. In addition, the calculated C‐index was 0.643 (Figure 2C,D).

### 3.2 Construction of the combination characteristics of ferroptosis and pyroptosis

**FIGURE 3:**
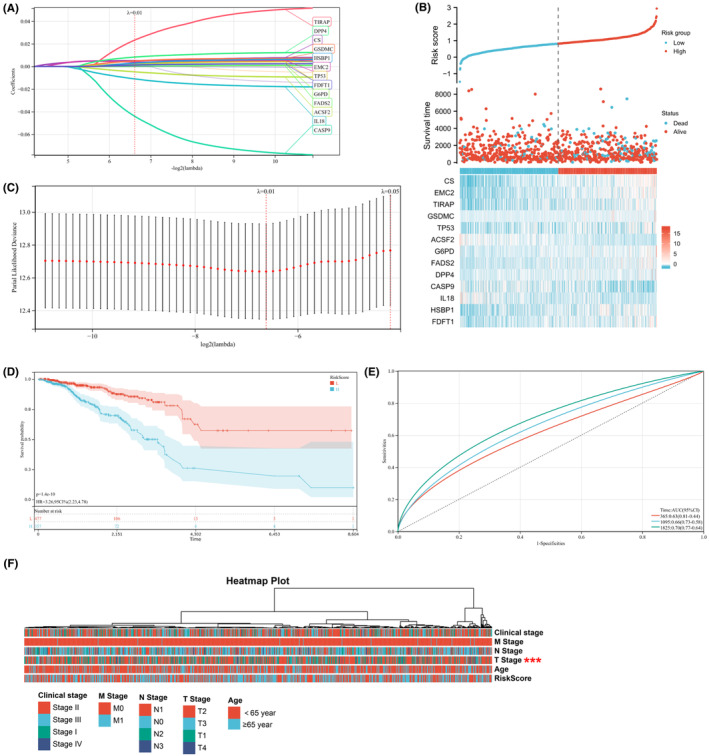
The predictive value of TCGA risk score in patient survival and different time. (A) Lasso‐cox regression analysis was used to construct the most effective prognostic indicators. (B) Prognostic Hub gene expression and survival after grouping according to risk score. (C) Lasso‐cox regression lambda coefficients. (D) K‐m test was used to analyse the survival curve of patients in high‐ and low‐risk group. (E) ROC curve was used to analyse the predictive value of risk score for 1‐, 3‐ and 5‐year survival. (F) Relationship between risk score and clinical data of breast cancer patients. *** indicates that the number of patients with high stage in the high‐risk group is higher than that in the low‐risk group.

LASSO regression analysis was used to develop a novel feature, considering the 17 prognostic genes described above, 13 genes were identified as the best variables. CS, EMC2, TP53, ACSF2, G6PD, FADS2, DPP4, HSBP1 and FDFT1 are ferroptosis genes, and the other four genes (TIRAP, GSDMC, CASP9 and IL18) are pyroptosis genes. The risk score for this new feature is, CS EMC2 + 0.0233179277462291 + 0.00395267450337161 * 0.00514170246740766 * * TIRAP GSDMC +0.00256300641149397 + 0.0048767629875963 * * TP 53–0.00478371479023051 * ACSF2 FADS2 G6PD e‐05 + 7.31104820070831 + 0.00166183499036533 * * * DPP4‐0.00827923024207522 + 0.044098216896 CASP9‐3227 * 0.0110266707194756 * IL18 FDFT1 HSBP1 + 0.00125703331243059 + 0.0029700094960144 * *. MRS was used as a cutoff to divide the BC samples into two groups, with 381 high‐risk cases and 365 low‐risk cases in the TCGA dataset (Figure 3A–C). Subsequently, the Kaplan–Meier analysis revealed worse OS in the high‐risk group (Figure 3E). The AUC of the one‐, three‐ and five‐year ROC were 0.63, 0.66 and 0.70, respectively (Figure 3E). In addition, the Wilcoxon signed‐rank test together with the chi‐square test indicated differences in the clinicopathological characteristics of the T stage between the risk groups (Figure 3F).

### 3.3 Verification of risk score of the TCGA database by GEO data

**FIGURE 4:**
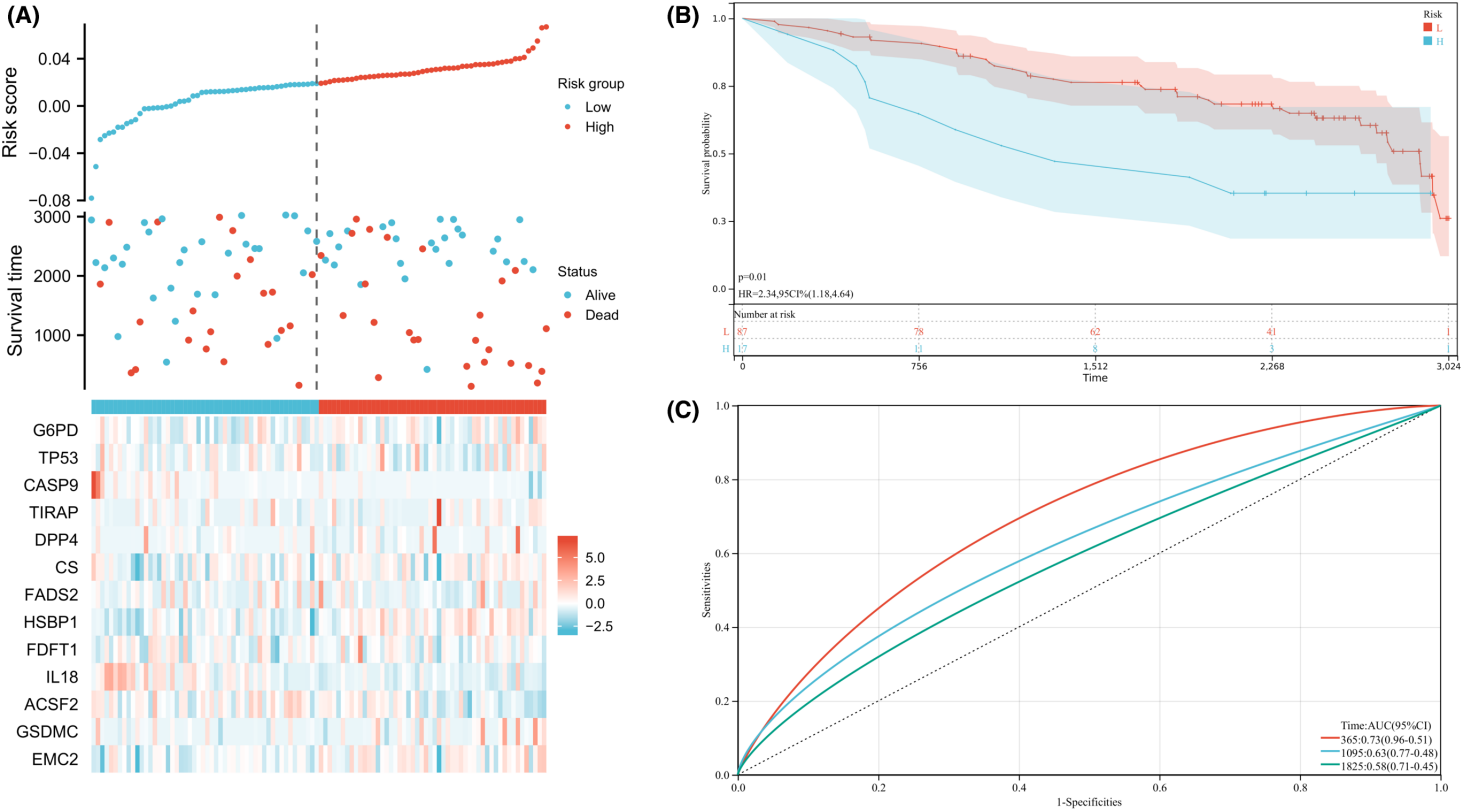
GEO was used to verify the predictive value of risk score in patient survival and different time. (A) Prognostic Hub gene expression and survival after grouping according to risk score. (B) K‐m test was used to analyse the survival curve of patients in high‐ and low‐risk group. (C) ROC curve was used to analyse the predictive value of risk score for 1‐, 3‐ and 5‐year survival.

BC data from the Gene Expression Omnibus (GEO) were used for risk score validation. GEO data were subjected to LASSO regression analysis to calculate patients' risk scores with similar regression coefficients (Figure 4A). This was followed by a KM survival analysis for risk model assessment. The high‐risk group had poorer OS than the low‐risk group (Figure 4B). An OS prediction model was constructed based on the ROC curves (Figure 4C).

### 3.4 Relationship between risk score and immune invasion in BC

**FIGURE 5:**
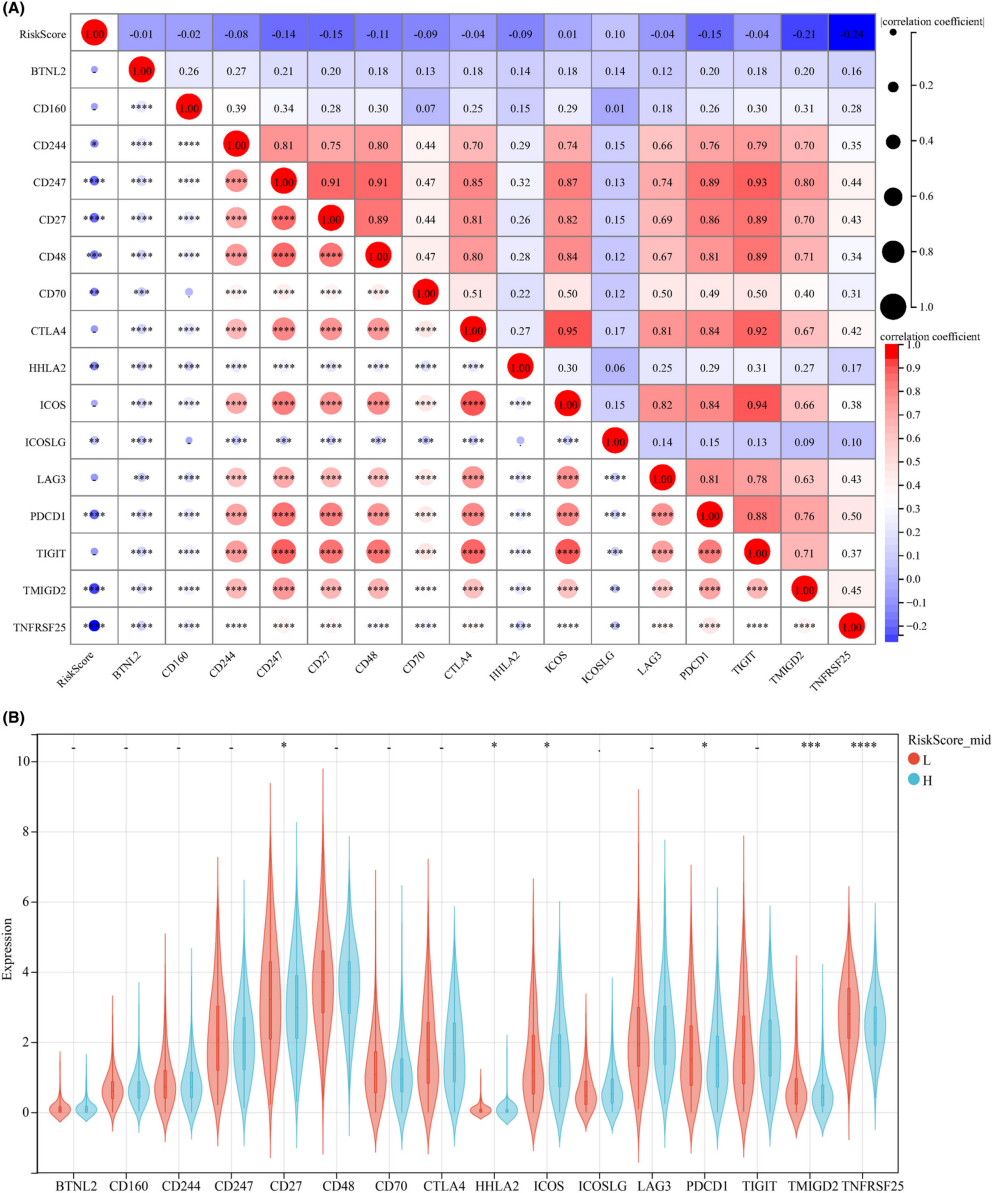
Relationship between risk score and immune checkpoint in breast cancer patients. (A) Correlation analysis between risk score and immune checkpoint in breast cancer patients. (B) Expression levels of immune checkpoint genes in patients with high and low risk of breast cancer. * denotes *p* < 0.05.

**FIGURE 6:**
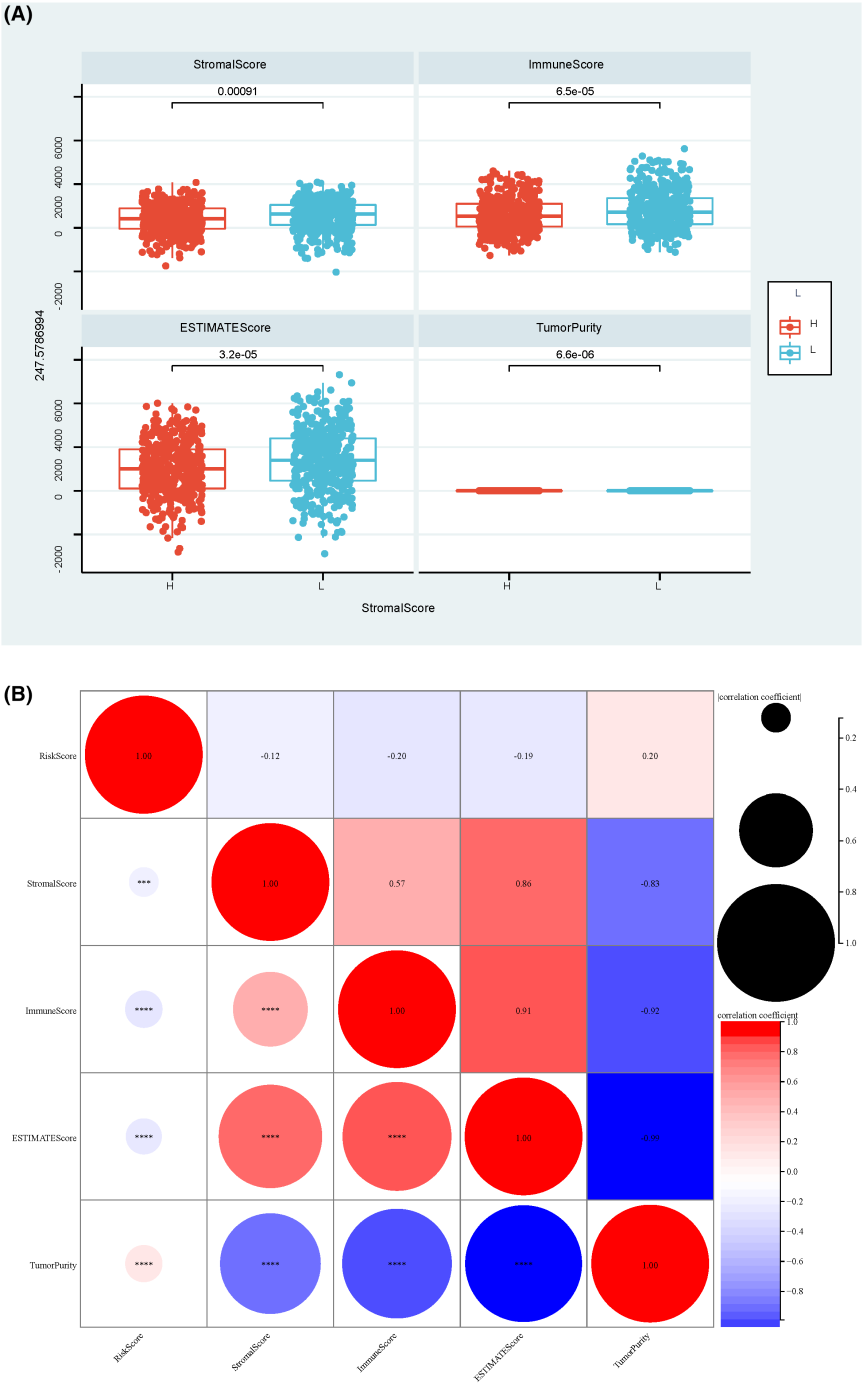
Relationship between risk score and ESTIMATE score in breast cancer patients. (A) Comparison of ESTIMATE score in breast cancer patients with high and low risk. (B) Correlation analysis between risk score and ESTIMATE score in breast cancer patients. * denotes *p* < 0.05; ** denotes *p* < 0.01; *** denotes *p* < 0.001; **** denotes *p* < 0.0001.

We employed ssGSEA to calculate the TCGA immune score, aiming at a deeper exploration of the association between risk score and immune status. The associations between risk scores and immune checkpoints (IMCP) were also investigated. The risk score was most strongly correlated with CD274, CD27, CD48, PDCD1, TMIGD2 and TNFRSF25. Further analysis showed that the high‐risk group presented remarkably higher CD27, ICOS, PDCD1, TMIGD2 and TNFRSF25 levels but remarkably lower HHLA2 levels relative to the low‐risk group. Therefore, it can be speculated that CD27, PDCD1, TMIGD2 and TNFRSF25 may participate in tumour immunity by regulating IMCPs (Figure 5A,B). We also calculated the number of BC immune cells and immune scores using the ESTIMATE algorithm. The results revealed a significantly lower Stroma Score, Immune Score and ESTIMATE Score but higher purity in the high‐risk group than in the low‐risk group. Correlation analysis found that the Stroma Score, Immune Score and ESTIMATE Score presented a negative relationship with the risk Score, while the Canine Purity presented a positive relationship (Figure 6A,B).

### 3.5 Immune microenvironment comparison between two risk groups

**FIGURE 7:**
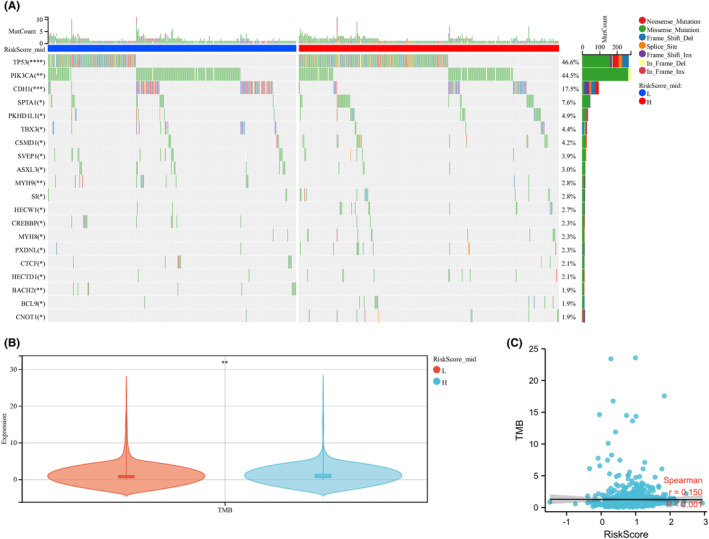
Association between risk score and TMB in breast cancer patients. (A) OncoPrint of frequently mutated genes in high‐risk and low‐risk subgroups. (B) Levels were compared between high—and low‐risk subgroups. (C) Correlation between risk score and TMB in breast cancer patients. ** denotes *p* < 0.01.

This study focused on analysing the association between the risk score and TMB and found differences in somatic TMB between the two groups (Figure 7A). The high‐risk group exhibited a higher TMB than the low‐risk group (Figure 7B). Correlation analysis revealed a positive association (Figure 7C).

### 3.6 BC classification based on gene association with ferroptosis and pyroptosis

**FIGURE 8:**
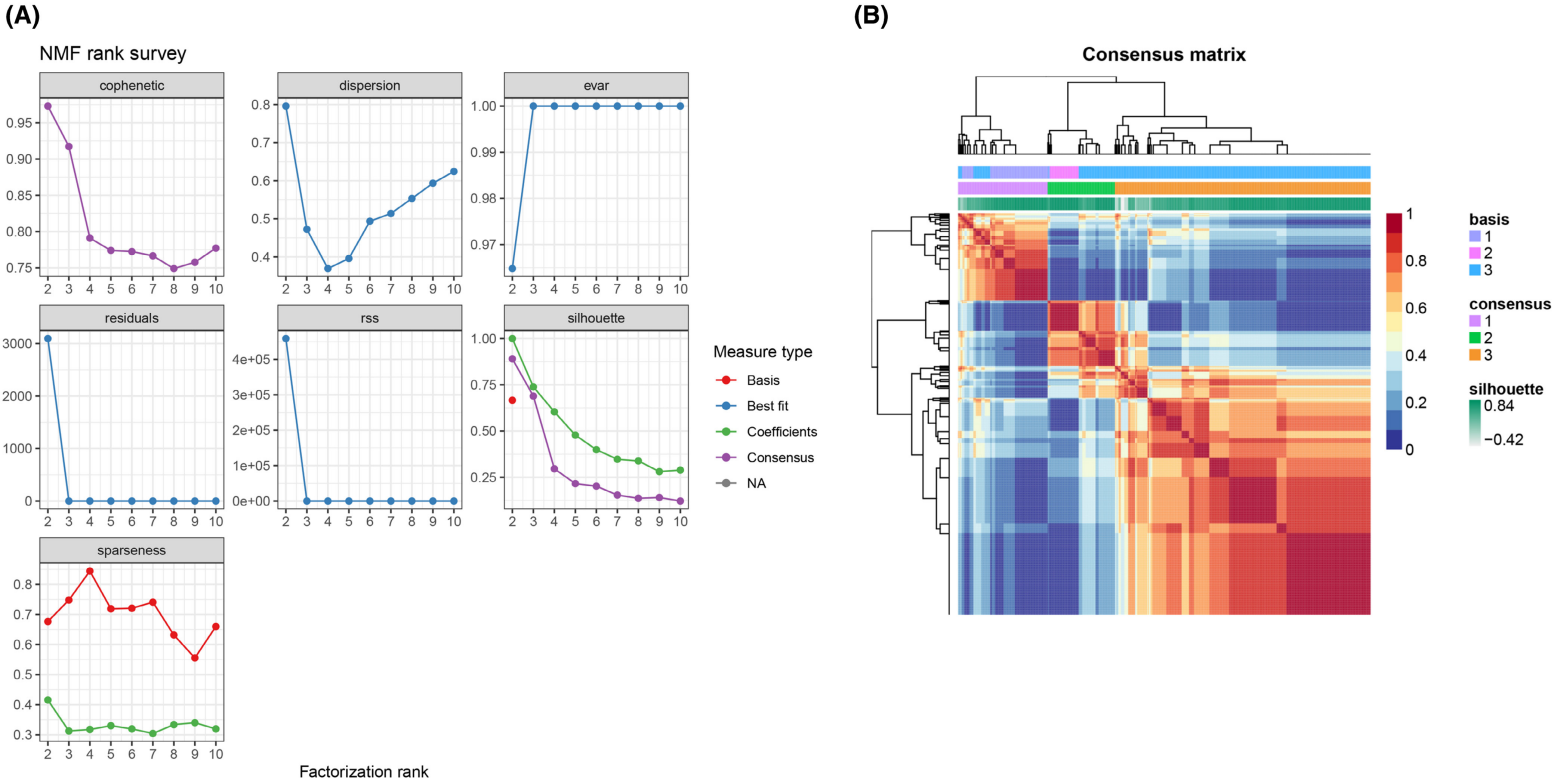
Using NMF to subtype breast cancer. (A) The NMF clustering gives the corresponding co‐occurrence correlation coefficients between 2 and 10 for *k* values. (B) NMF clustering of 17 genes related to ferroptosis and pyroptosis was used.

The TCGA RNA‐seq dataset contributed to the gene expression profiles of 1098 samples and the gene matrix related to ferroptosis and pyroptosis. Seventeen gene matrices related to ferroptosis and pyroptosis were screened by survival analysis. We then performed NMF analysis to classify the BC samples into three distinct clusters (C1, C2 and C3), which aimed to decompose the original matrix into two nonnegative matrices to identify the potential characteristics exhibited by the gene expression profiles. We employed a comprehensive correlation coefficient to confirm the optimal k value and then set the optimal cluster number to *k* = 3 (specifying the two subclasses as C1, C2 and C3). Specifically, when *k* = 3, the consensus matrix heat map maintained distinct and sharp boundaries, revealing steady clusters in the sample (Figure 8A,B).

### 3.7 Association between BC subgroups and immune cell infiltration

**FIGURE 9:**
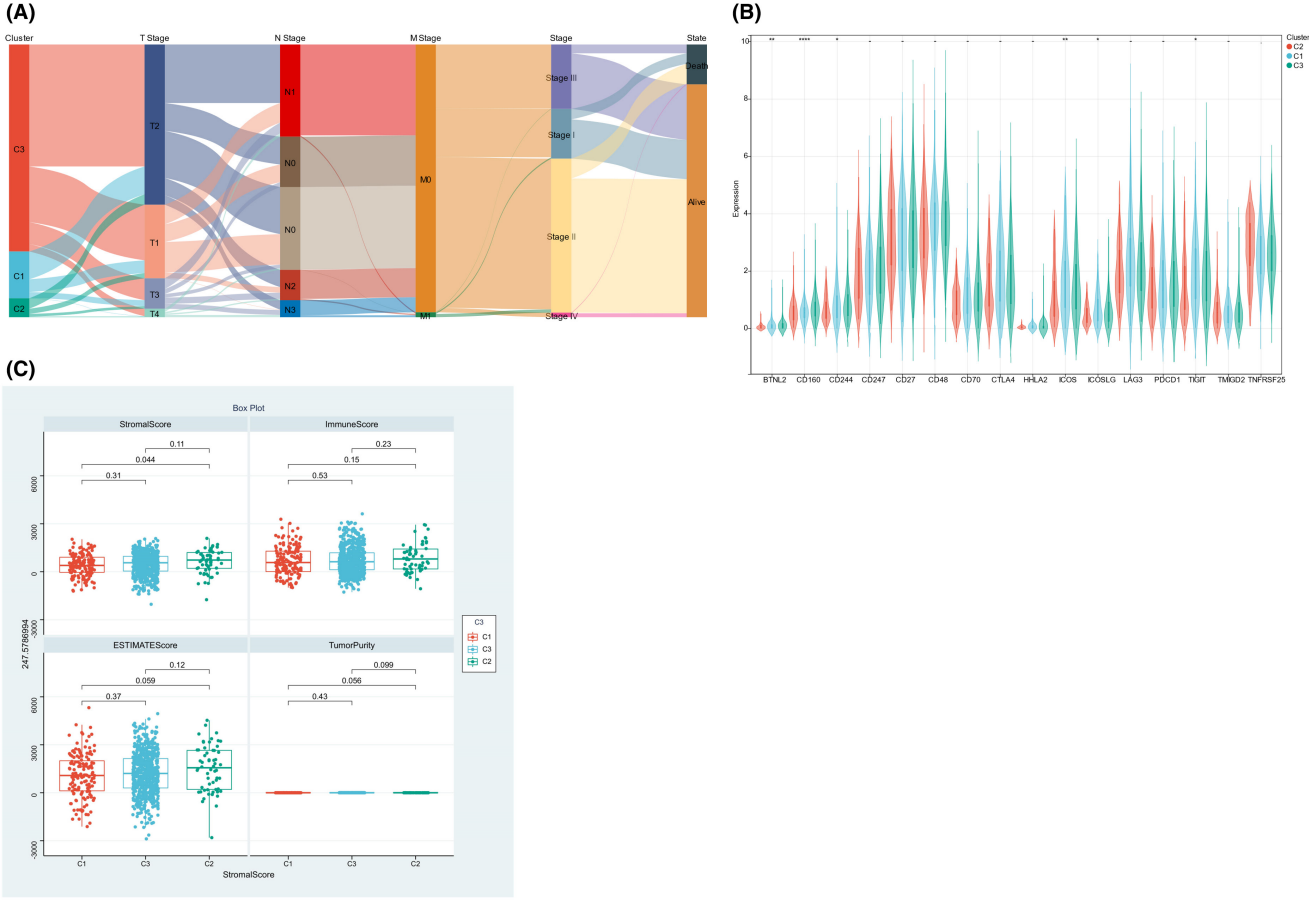
Association between breast cancer subgroups and immune cell infiltration. (A) Sankey diagram was used to show the baseline data distribution of patients. (B) Comparison of breast cancer subgroup and immune checkpoint expression. (C) Comparison of ESTIMATE score in breast cancer subgroups.

After grouping the TCGA dataset using NMF, we obtained three clusters (Figure 9A), followed by analysing the association of IMCP with the ESTIMATE score of subgroups of patients, finding the differentially expressed BTNL2, CD160, CD244, ICOS, ICOSLG and TIGIT in the C1, C2 and C3 clusters (Figure 9B). In addition, further analysis found a higher stroma score and ESTIMATE Score in the C2 cluster relative to the C1 cluster, and a higher Purity in the C2 cluster relative to the C3 cluster (Figure 9C).

## 4 DISCUSSION

BC is a common malignant tumour in females and presents a poor prognosis.^18^ New biomarkers can predict the prognosis of cancer patients.^19^ Ferroptosis and pyroptosis are two new cell death modes that have been shown to affect tumour progression.^20^ However, few studies have explored the efficacy of combining these two modes in BC.

In this study, we identified 60 ferroptosis genes and 33 pyroptosis genes associated with ferroptosis from review articles and published experiments. Relevant analyses were mainly used to identify prognostic genes. Finally, we constructed a prognostic model consisting of 13 genes to predict the prognosis and validated it in the external cohort of GSE42568. A C‐index of 0.612–0.674 indicated a predictive effect on short‐term prognosis. Ferroptosis and pyroptosis potentially influence BC, and it is possible to characterize their prognostic expression.

Ferroptosis is an iron‐dependent cell death mode that is different from other cell death modes (apoptosis, autophagy and necrosis) from biochemical, morphological and genetic perspectives.^21^ This process features an iron‐dependent elevation in reactive oxygen species (ROS), cell membrane thickening and mitochondrial volume reduction.^22^ Recent reports have identified several factors that induce ferroptosis, including Erastin, Sulfasalazine, RSL3 and Cysteine starvation.^23^ Moreover, GPX4, P53, HSPB1, CISD1, CHAC1, CARS, SLC7A11 and TFR1 are closely associated with ferroptosis.^21^ In a study by Ma et al.,^24^ the combination of siramesine and lapatinib induced ferroptosis by reducing iron transporter (FPN) expression and increasing transferrin expression. Pyroptosis is a novel form of programmed cell death that exerts a dual action in various cancers.^25^, ^26^, ^27^ It has been reported^28^ that the PD‐L1‐mediated transition from apoptosis to pyroptosis promotes tumour necrosis, assists in the growth of tumour promoters and hinders antitumor immunity. In addition, Gao et al.^29^ found that increased expression of GSDMD promotes NSCLC evasion of the innate immune response. Another study showed^30^ that GSDME exerted tumour‐suppressive effects by activating pyroptosis and enhancing antitumor immunity. However, the number of reports that have investigated the roles of ferroptosis and pyroptosis in BC is small and differs from that of this study. For example, some studies have investigated the clinical features and prognostic value of five programmed cell death (PCD) pathways (apoptosis, ferroptosis, necroptosis, pyroptosis and autophagic cell death) in BC. Finally, the status of the ferroptosis pathway was found to be significantly correlated with the clinical outcome and intratumoral heterogeneity of BC, and the expression of NDUFA13 was identified as a positive biomarker of ferroptosis pathway activation in patients with BC.^31^ This study focused on the roles of ferroptosis and pyroptosis in BC and analysed the relationship between ferroptosis and immune infiltration. Other researchers have carried out Transmembrane protein 65 (TMEM65) pan‐cancer studies, and the results found TMEM65 to be associated with certain pathways (TGF beta signalling, TNFA signalling, hypoxia, pyroptosis,^32^ DNA repairing, autophagy, ferroptosis, ferroptosis and ferroptosis) in BC. In contrast, this study focuses on TMEM65 and confirms the role of ferroptosis and pyroptosis in BC from a different perspective. Another study comprehensively examined the role of programmed cell death (PCD), including ferroptosis, pyroptosis and necroptosis in BC.^33^ The method described in this manuscript is partially similar to that described in this manuscript, but the scope of the study is broader and has not been verified in another database. However, the results also indicated that ferroptosis, pyroptosis, the immune microenvironment, and BC are directly and inextricably linked.

This study systematically investigated the expression and prognostic value of 60 ferroptosis and 33 pyroptosis genes in BC. Univariate Cox analysis revealed that more than 12 ferroptosis genes affected the OS of BC patients, whereas only 5 pyroptosis genes were associated with OS in BC patients. Ferroptosis and pyroptosis play potential roles in BC, and it is possible to characterize their prognostic expression. To further explain the effects of ferroptosis and pyroptosis genes on BC prognosis, a prognostic model was constructed using least absolute shrinkage and selection operator (LASSO). Finally, a prognostic model was constructed for 13 genes (CS, EMC2, TP53, ACSF2, G6PD, FADS2, DPP4, HSBP1, FDFT1, TIRAP, GSDMC, CASP9, IL18) was constructed and verified using external data. This risk model could predict BC survival. In addition, most of the genes in our model were strongly associated with cancer timing. For example, CS is present in almost all cells capable of oxidative metabolism, and Cai^34^ et al. indicated the effect of abnormal CS expression on prostate cancer progression. In addition, other studies have found^35^ that CS targets glycolysis and the cancer stem cell phenotype to trigger invasive triple‐negative BC cells. TP53 encodes the tumour protein p53, a tumour suppressor capable of inhibiting cell division and proliferation. Research has confirmed this as a biomarker for predicting BC prognosis.^36^ G6PD is an essential enzyme that produces NADPH for the maintenance of reduced glutathione levels and the removal of ROS. Previous studies^37^, ^38^ found that high G6PD expression leads to a worse prognosis in patients with bladder and colon cancers. FADS2 is overexpressed in colorectal cancer and promotes cancer cell proliferation by increasing the metabolism of oncogenic molecules related to colorectal tumorigenesis.^39^ DPP4 (CD26) is a cell surface protein that affects tumour biology and immune system regulation. Studies^40^ have revealed high DPP4 expression in patients with prostate cancer, pancreatic cancer and BC. Moreover, DPP4 inhibition accelerates epithelial/mesenchymal transition and metastasis in BC via the CXCL12/CXCR4/mTOR axis.^41^ Downregulation of FDFT1 promotes malignant progression and worsens the prognosis of CRC,^42^, ^43^ whereas high FDFT1 expression results in enhanced invasion of prostate cancer.^44^ The Gasdermin protein family is the most significant regulator of pyroptosis. Recent studies have shown^28^ that GSDMC and PD‐L1 can cause BC tissue necrosis by transforming apoptosis into pyroptosis under hypoxic conditions. In addition, Sun et al.^45^ revealed that high expression of GSDMC leads to poor prognosis and tumour immune invasion in BC. CASP9 is an essential therapeutic target for various apoptosis‐related diseases, including cancer.^46^ IL‐18 plays a dual role in cancer by promoting tumour development, progression, invasion, migration and metastasis.^47^ The roles of CASP9 and IL‐18 in BC have been extensively reported. However, there are few reports on the expression of EMC2, ACSF2, HSBP1 and TIRAP in BC.

Recent studies have shown that ferroptosis and pyroptosis are associated with antitumor activity. Tumour cells that undergo these two processes are capable of recruiting tumour suppressor immune cells and enhancing antitumor immunity.^48^ Therefore, in this study, we analysed the relationship between the risk model and immune infiltration. Previously, we examined the association between risk score and IMCP genes. In our study, we found that the high‐risk group had markedly higher CD27, ICOS, PDCD1, TMIGD2 and TNFRSF25 levels and lower HHLA2 levels than the low‐risk group. Therefore, CD27, PDCD1, TMIGD2 and TNFRSF25 may participate in tumour immunity by regulating IMCPs. Subsequently, we also found that the high‐risk group presented remarkably lower Stroma Score, Immune Score and ESTIMATE Score, but significantly higher purity relative to the low‐risk group, indicating a relationship between the risk model and immune infiltration. TMB can predict the efficacy of IMCP blockade and is a biomarker for identifying patients benefiting from immunotherapies specific to various cancer types. In our results, the high‐risk group had a higher TMB than the low‐risk group, indicating that the large number of mutated genes in the high‐risk group resulted in a poor prognosis. As the current immunotherapy for BC is still in its infancy, patients with a poor prognosis may benefit from a high TMB score and a larger number of mutated genes.

This study focused on analysing the relationship between ferroptosis and pyroptosis genes and the prognosis and immune invasion of BC using two databases and successfully constructed a prognostic model. However, this study had two limitations. (1) Since no clinical samples were collected, the mutual verification between the two datasets could not prove the universality of the risk model. (2) Basic research was not conducted; hence, it is still unclear whether the genes in the risk model have the same expression in cell lines or animal models and possess a regulatory mechanism. Therefore, more experiments should be conducted to improve the conclusions of this study.

In conclusion, we have successfully identified the combined features of ferroptosis and pyroptosis in BC, which are closely related to prognosis, clinicopathological features, immune features, chemotherapy effects and immunosuppressive molecules. However, owing to the lack of further in vitro and in vivo verification experiments, the evidence level of many results is insufficient, and the role and relationship of various molecular pathways in BC cannot be explained in detail and more directly. Therefore, in future research, we should focus on the verification of the research results in this manuscript, in cell and animal experiments, and further improve the detailed and specific mechanism of action.

## AUTHOR CONTRIBUTIONS

**Lili Zhou:** Data curation (equal); formal analysis (equal); software (equal); writing – original draft (equal). **Chinting Wong:** Writing – review and editing (equal). **Yang Liu:** Resources (equal). **Wenyan Jiang:** Validation (equal). **Qi Yang:** Conceptualization (lead).

## FUNDING INFORMATION

This research was supported by the National Natural Science Foundation of China (ID:82272059) and the Finance Department of Jilin Province (ID: JLSWSRCZX2020‐069).

## CONFLICT OF INTEREST STATEMENT

The authors confirm that there are no conflicts of interest.

## Data Availability

The data that support the findings of this study are available from the corresponding author upon reasonable request.
